# Local Trust in Internet of Things Based on Contract Theory

**DOI:** 10.3390/s22062393

**Published:** 2022-03-20

**Authors:** Georgios Fragkos, Cyrus Minwalla, Jim Plusquellic, Eirini Eleni Tsiropoulou

**Affiliations:** 1 Department of Electrical and Computer Engineering, University of New Mexico, Albuquerque, NM 87131-0001, USA; gfragkos@unm.edu (G.F.); jplusq@unm.edu (J.P.); 2 Financial Technology Research, Bank of Canada, Ottawa, ON K1A 0G9, Canada; cminwalla@bank-banque-canada.ca

**Keywords:** Bayesian model, contract theory, crowdsourcing, Internet of Things, PeerTrust, reinforcement learning

## Abstract

Autonomous trust mechanisms enable Internet of Things (IoT) devices to function cooperatively in a wide range of ecosystems, from vehicle-to-vehicle communications to mesh sensor networks. A common property desired in such networks is a mechanism to construct a secure, authenticated channel between any two participating nodes to share sensitive information, nominally a challenging proposition for a large, heterogeneous network where node participation is constantly in flux. This work explores a contract-theoretic framework that exploits the principles of network economics to crowd-source trust between two arbitrary nodes based on the efforts of their neighbors. Each node in the network possesses a trust score, which is updated based on useful effort contributed to the authentication step. The scheme functions autonomously on locally adjacent nodes and is proven to converge onto an optimal solution based on the available nodes and their trust scores. Core building blocks include the use of Stochastic Learning Automata to select the participating nodes based on network and social metrics, and the formulation of a Bayesian trust belief distribution from the past behavior of the selected nodes. An effort-reward model incentivizes selected nodes to accurately report their trust scores and contribute their effort to the authentication process. Detailed numerical results obtained via simulation highlight the proposed framework’s efficacy and performance. The performance achieved near-optimal results despite incomplete information regarding the IoT nodes’ trust scores and the presence of malicious or misbehaving nodes. Comparison metrics demonstrate that the proposed approach maximized the overall social welfare and achieved better performance compared to the state of the art in the domain.

## 1. Introduction

The convergence of ubiquitous networking, cloud computing, and embedded intelligence has led to the rise of edge computing and the Internet of Things (IoT). Applications for IoT range from home to industrial automation, from local sensor network to vehicle-to-vehicle (V2V) communications. New challenges emerge as these networks evolve from local, constrained environments to large, heterogeneous ecosystems, where the cardinality and capability of individual nodes is always in flux.

Maximum utility is derived if such ecosystems become capable of sharing sensitive information, such as healthcare or payments data. To achieve this, the nodes must be able to participate in a trust framework that enables secure communication channels. While centralized approaches, such as standards and certificates, solve the problem, they require an established infrastructure to function effectively. A centralized authority is needed to issue and disseminate certificates, prior registration of all participating devices is required, and all nodes must agree on a common communications protocol. Such an approach is unsuited for large, heterogeneous networks where nodes vary greatly in compute power, communications protocols and standards compliance, as certain nodes may not be registered or may be incapable of participating due to missing hardware or insufficient compute power.

Decentralized approaches are far more effective in this regard, the most promising of which is the crowd-sourced trust model, where trust between two IoT devices is derived from transient neighbouring nodes available at that point in time, despite their hardware configuration, compute power and protocol support.

Each device needs only to store a trust score, a normalized floating point value. The score can be bootstrapped to a default value for new devices. No special hardware or compute function is required to maintain or update the score, as integrity in the score is established through the crowd-sourcing mechanism. Robustness of the method is enhanced if differences in configuration and capability contribute to the derivation of the final trust score.

Presented in this work is a contract-theoretic mechanism based on incentives for building trust between two devices (termed Alice and Bob) operating in a large, heterogeneous network of IoT nodes. The proposed approach introduces a novel trust model and trust-based management features at the protocol level. The model derives trust in an ad-hoc fashion by crowd-sourcing locally adjacent nodes while maintaining robustness against malicious behavior. There is no reliance on cryptographic primitives or connection to a central authority.

The approach is distinct from traditional blockchain solutions in that the final outcome (consensus) is local to Alice and Bob and not a common state shared with all nodes in the system. Furthermore, by relying on simple metrics that all nodes possess, the model establishes independence from the underlying hardware and communications architecture, and is therefore compatible with a wide variety of applications. Crucially, the approach is compatible across network boundaries and can bind cross-network devices in a common trust framework, a property not commonly seen in other approaches. Highlights of the scheme are as follows:

1. Use of Stochastic Learning Automata (SLA), to select crowd-sourced nodes in an autonomous and distributed manner. In particular, the selection at every iteration of the utilized Reinforcement Learning (RL) algorithm is probabilistically reinforced with respect to the network characteristics, such as delay and congestion, and social characteristics, such as trust scores.
2. Introduction of a Bayesian trust model to probabilistically estimate the nodes’ trust scores in the absence of complete information in a realistic Internet of Things environment.
3. Formulation of a novel PeerTrust protocol coupled with Bayesian adverse selection to model Alice’s personalized belief of node trust levels despite the nodes’ potential false individual reports of trustworthiness.
4. Introduction of a novel contract-theoretic scheme based on the theory of labor economics that operates under the scenario of information asymmetry, yet incentivizes nodes to contribute effort and receive rewards corresponding to actual trust levels. Ergo, the trust model operates with incomplete information, where the optimal pairing of effort and reward represents the contract.
5. Formulation of payoff functions for Alice, Bob and all participating nodes, which are maximized under certain constraints that hold true within the IoT network. The non-convex optimization problem is transformed into a convex form, with the optimal efforts-reward pairs determined accordingly. An extensive numerical and comparative evaluation to demonstrate the operation and efficiency of the proposed framework.

### Related Work

Decentralized trust models are an emerging topic of interest in IoT environments. In [1], the authors present a novel framework based on the evolutionary game theory and Lyapunov optimization to qualitatively study and prove the stability and validity of the proposed IoT trust management scheme. A similar approach is followed in [2], where blockchain technology along with the Evolutionary Combination game-theoretic rule [3] are adopted.

Specifically, the authors formulate a robust decentralized trust management scheme is introduced that mitigates the impact of malicious nodes that send false trust scores. In [4,5], the authors adopt a blockchain data structure to enhance the IoT capabilities and a decentralized trust management framework is introduced to enable dynamic access control policies. A K-means-based approach is proposed in [6], which assesses the trustworthiness of the IoT nodes by sharing certain information among them.

In [7,8], the authors propose a fuzzy security protocol for trust management and a Beta distribution-based trust technique for information-centric-networks aiming at detecting on-off attacks or malicious nodes. The CTRUST model is proposed in [9] to study the effects of trust decay and maturity in a decentralized collaborative download application.

Moreover, the authors in [10] introduce a quantifiable trust assessment model for IoT services based on the K-means and Support Vector Machine (SVM) algorithms to efficiently extract trust features from raw data and perform trustworthiness-based decision-making. The role of Artificial Social Intelligence (ASI) in the management of the IoT-enabled social relationships is discussed in [11], where the authors maintain that trust management is vital among social IoT devices for social clustering and community detection.

Two blockchain-based trust management schemes for IoT nodes are adduced in [12,13]. The first one stores each node’s trust scores in the blockchain via hashed transactions and shares them with other nodes within the network, while the latter one utilizes the decentralized architecture to evaluate the process’s trustworthiness and guarantee the satisfaction of IoT nodes’ energy constraints regarding trust computation. The integration of a dynamic trust management model based on a hybrid environment consisting of industrial communities is demonstrated in [14].

In [15], the cumulative trust concept models the trust management in IoT nodes by measuring the packet drop and data rates among them. An alternative trust scheme is introduced in [16], where a recommendation filtering algorithm is proposed based on the Bayesian inference model to mitigate bad recommendations. In [17], a context-based trust evaluation system is presented to mitigate service-oriented attacks based on the Naive Bayesian method.

Literature is limited on efforts to model a trust management scheme that does not have perfect knowledge of the IoT environment’s characteristics, where nodes may act in a malicious manner by exploiting the incomplete information setting to lie about their own trust levels or collude with other nodes. The problem difficulty increases dramatically in an ‘offline’ setting, where an ad-hoc network has no connectivity to a remote back-end (source or sink). A representative application example is a Wireless Mesh Network (WMN) that reliably connects multiple heterogeneous IoT nodes to a centralized Bank for transactions processing and verification.

The IoT nodes enable automated e-payments by adopting wearable e-payment methods, e.g., via smartwatches, rather than carrying traditional credit cards. However, a common real-world challenge is that the IoT nodes may not have Internet connectivity. As a consequence, the respective IoT nodes form an offline ad-hoc network that is responsible for transactions processing and verification by creating a secure channel between any pair of nodes (termed Alice and Bob) based on offline trust management. Towards achieving this goal, a scheme is required to incentivize IoT nodes in reporting their trust levels truthfully and contributing effort to build trust between Alice and Bob.

The rest of the paper is organized as follows. Section 2 introduces the system model, the Bayesian belief scheme, and the PeerTrust model, while Section 3 elaborates on the contract-theoretic interactions among the IoT nodes. Section 4 presents the IoT nodes’ selection by Alice based on the SLA model. Finally, the numerical and comparative evaluation is presented in Section 5 and Section 6 concludes the paper.

## 2. System Model

An offline IoT environment is considered, consisting of |C| IoT nodes that are willing to participate in the crowd-sourcing process to facilitate the secure interaction between Alice and Bob. Their corresponding set is denoted as C={1, …,c, …, |C|}. $C_{A}^{t}$ denotes the set of nodes selected by Alice at time slot *t* by utilizing Reinforcement Learning (Section 4) to provide to her identification information (effort) related to Bob. Alice’s distance from each node *c* is $d_{c}^{t}\left[m\right]$ and the established communication link among them experiences a normalised congestion $cr_{c}^{t}\in \left[0,1\right]$ due to the exchange of information in a peer-to-peer manner. A summary of the used key notations is provided in Table 1.

### 2.1. The Concept of Contract

To establish a secure channel with Bob, Alice needs to initiate a mobile crowdsourcing process, where each selected IoT node $c\in C_{A}^{t}$ provides some effort $e_{A,c}^{t}\in \left[0,1\right]$ in time slot *t*. In the trust model, this effort can be represented as the contribution of unique identification information about Bob provided by the selected nodes. However, in order for Alice to ensure the cooperation from the IoT nodes regarding Bob’s authentication, she utilizes an incentivization mechanism by providing each node an appropriate personalized reward $r_{A,c}^{t}\in \left[0,1\right]$, such as a monetary payment or an increment to their recorded absolute trust levels to compensate them for their effort.

In our analysis, the effort $e_{A,c}^{t}\in \left[0,1\right]$ invested by each selected IoT node *c* to facilitate Alice’s interaction with Bob and the corresponding received reward $r_{A,c}^{t}\in \left[0,1\right]$ are considered as normalized variables on the interval [0,1]. In a real-life scenario, those variables can be mapped to realistic metrics, e.g., the amount of unique identification information for Bob that each selected IoT node offers to Alice as its effort, and respective changes in the trust levels of the nodes made by Alice as a reward.

Intuitively, a labor economics-based relationship is formulated between Alice and the selected IoT nodes, where the more identification data an IoT node provides to Alice, the more its trust level will be increased as a reward for its effort. The provided reward from Alice is used to update the node’s trust level (see Section 2.3). The pair of (effort, reward) constitutes a contract among Alice and each selected IoT node, denoted as $\left(e_{A,c}^{t},r_{A,c}^{t}\right)$.

Accordingly, payoff functions are formulated for both Alice and the selected IoT nodes (see Section 2.4) and an optimization problem is formulated where the optimality for the aforementioned payoff functions is guaranteed. The optimization problem is solved and the optimal IoT nodes’ efforts are determined.

### 2.2. Bayesian Trust Belief

In a realistic offline IoT environment, Alice has incomplete information regarding the quality of effort $e_{A,c}^{t}$ that each selected node can contribute to help with Alice establishing a secure channel with Bob. For instance, in the aforementioned WMN example, Alice stores offline her own belief regarding the potential quality of unique identification data about Bob that each IoT node can offer her, since she cannot be certain if the provided information will help her to successfully authenticate Bob.

This belief is updated throughout the time horizon and exploited when Alice acts as an RL agent and selects a subset of IoT nodes to verify her transaction with Bob. Thus, we utilize the concept of Bayesian trust belief $\mu_{A,c}^{t}\in \left[0,1\right]$, of Alice regarding another IoT node *c*, c∈C, at a specific time slot *t*. Towards determining the trust belief of each IoT node, we deploy a Bayesian model featuring the theory of adverse selection [18] and Bayesian updating of belief [19].

Initially (t=0), all IoT nodes have the same prior trust belief distribution, i.e., $\mu_{A,c}^{0}=\mu_{0},\forall c,\in C$, regarding the contribution that each node can provide to the establishment of secure interactions by others. Each node can either provide a high or low contribution to the crowdsourcing process, with probabilities $a_{h}$ and $a_{l}$, respectively, where $0<a_{l}<a_{h}<1$. Given the selection of the set of IoT nodes $C_{A}^{t}$ by Alice at a time slot *t*, each IoT node’s $c\in C_{A}^{t}$ contribution is evaluated as satisfactory or not if it agrees to participate in the crowdsourcing process or not, respectively.

History is constructed for every IoT node throughout the time horizon, where $S_{c}^{t}$ and $F_{c}^{t}$ denote the number of times until time slot *t* that node *c* has contributed or not in the crowdsourcing process in a satisfactory manner, respectively. Alice’s posterior trust belief regarding every other IoT node *c* is given as follows.

(1) $$\mu_{A,c}^{t}=\frac{\mu_{0}a_{h}^{S_{c}^{t}}{\left(1-a_{h}\right)}^{F_{c}^{t}}}{\mu_{0}a_{h}^{S_{c}^{t}}{\left(1-a_{h}\right)}^{F_{c}^{t}}+\left(1-\mu_{0}\right)a_{l}^{S_{c}^{t}}{\left(1-a_{l}\right)}^{F_{c}^{t}}}.$$

**Theorem** **1.** *The posterior trust belief $\mu_{A,c}^{t}$ is an increasing function with respect to the positive $S_{c}^{t}$ and a decreasing function with respect to the negative evaluations $F_{c}^{t}$.*

**Proof** **of** **Theorem** **1.** We denote $\gamma =\mu_{0}a_{h}^{S_{c}^{t}}{\left(1-a_{h}\right)}^{F_{c}^{t}}$ and $\delta =\left(1-\mu_{0}\right)a_{l}^{S_{c}^{t}}{\left(1-a_{l}\right)}^{F_{c}^{t}}$. It holds true that γ,δ>0, as $\mu_{0},a_{h},a_{l}\in \left(0,1\right)$ and $S_{c}^{t},F_{c}^{t}>0,\forall c\in C,\forall t$. The first order partial derivatives of $\mu_{A,c}^{t}$ with respect to $S_{c}^{t}$ and $F_{c}^{t}$ are considered to examine the monotonicity of the posterior trust belief. Initially, we have $\frac{\partial \mu_{A,c}^{t}}{\partial S_{c}^{t}}=\frac{\gamma \delta \ln\left(\frac{a_{h}}{a_{l}}\right)}{{\left(\gamma +\delta \right)}^{2}}>0$, given that γ,δ>0 and $0<a_{l}<a_{h}<1$. Thus, $\mu_{A,c}^{t}$ is a strictly increasing function with respect to $S_{c}^{t}$. Similarly, we have $\frac{\partial \mu_{A,c}^{t}}{\partial F_{c}^{t}}=\frac{\gamma \delta \ln\left(\frac{1-a_{h}}{1-a_{l}}\right)}{{\left(\gamma +\delta \right)}^{2}}<0$. Thus, $\mu_{A,c}^{t}$ is a stricly decreasing function with respect to $F_{c}^{t}$. □

The physical meaning of Theorem 1 is that each IoT node gains an increasing posterior trust belief, if its contribution to the crowdsourcing process is evaluated as satisfactory over time, e.g., if each IoT node offers to Alice an adequate quality of unique identification data for Bob in the WMN application scenario. The posterior trust belief will be further used to enable the offline contract-theoretic interaction among Alice and the selected IoT nodes (see Section 2.4).

### 2.3. IoT Node Score—A PeerTrust Modeling

Each IoT node is characterized by a score $p_{c}^{t}$ that captures its private information regarding how frequently and how efficiently it has participated in the crowdsourcing process throughout the time horizon. In the WMN application scenario mentioned in Section 1, the score of an IoT node could reflect how often it assists in the creation of a secure transaction channel between Alice and Bob and the offline verification of the transactions by providing high-quality identification data for Bob to Alice. The score of an IoT node is based on the theory of score within PeerTrust [20], which is a peer-to-peer (P2P) reputation-based trust supporting framework.

Each IoT node’s score $p_{c}^{t}\in \left[0,1\right]$ is defined as follows:

(2) $$p_{c}^{t}=\frac{T_{c}}{\sum_{\forall c^{\prime }\in C}T_{c^{\prime }}}$$

where T(c) denotes the node’s trustworthiness. Note that in a real setting, the set *C* may be limited to nodes available locally as part of the ad-hoc network. Considering that each IoT node stores locally its own absolute trust score $p_{c}^{t}$, the system is bootstrapped by having each node transmit Wi-Fi beacon frames advertising its own absolute score. Therefore, other IoT nodes, e.g., Alice, is able to use those to compute the transmitting nodes’ relative scores, i.e., her personalized belief regarding the quality of the identification data coupled with the transmitted absolute scores.

Based on the PeerTrust model [20], four important factors are utilized to define the node’s trustworthiness: (a) the reward $r_{A,c}^{t}$ that an IoT node *c* receives from Alice at a certain time slot *t*, (b) the overall number of interactions that the IoT node has with Alice denoted as I(A,c), (c) the credibility factor of Alice expressed via its posterior Bayesian trust belief $\mu_{c,A}^{t}$, and (d) the interaction context factor $TF_{A,c}^{t}\in R_{0}^{+}$, which can be used to characterize the criticality and importance of interaction among Alice and Bob. Thus, the trustworthiness of each IoT node is defined as the weighted sum of the amount of satisfaction that IoT node *c* receives in each crowdsourcing interaction with Alice:

(3) $$T_{c}=\alpha \cdot \sum_{i=1}^{I(A,c)}r_{A,c}^{i}\cdot \mu_{c,A}^{i}\cdot TF_{A,c}^{i}$$

where α∈[0,1] is a normalized weighting factor. Equation (3) can be interpreted as the prediction of IoT node’s *c* likelihood of a satisfactory contribution in the crowdsourcing. For presentation purposes, we sort the nodes’ scores in an ascending order at a specific time slot *t*, i.e., $p_{1}^{t}<\cdots <p_{\left|C\right|}^{t}$.

### 2.4. Alice’s and Selected IoT Nodes’ Payoff

Each IoT node $c\in C_{A}^{t}$ is characterized by a payoff function $U_{c}^{t}\left(e_{A,c}^{t}\right)$ at a specific time slot *t*, which represents its benefit from the reward $r_{A,c}^{t}$ offered by Alice, while considering its personal cost to provide the effort $e_{A,c}^{t}$ to Alice. The node’s payoff is defined as follows

(4) $$U_{c}^{t}\left(e_{A,c}^{t}\right)=p_{c}^{t}\cdot q\left(r_{A,c}^{t}\right)-e_{A,c}^{t}$$

where $q(r_{A,c}^{t})$ is the evaluation function of the received reward $r_{A,c}^{t}$. The evaluation function is continuous, strictly increasing, and concave with respect to the received reward, i.e., $q\left(0\right)=0,\;q^{\prime }\left(r_{A,c}^{t}\right)>0,\;q^{\prime \prime }\left(r_{A,c}^{t}\right)<0$. For demonstration purposes and without loss of generality, we consider $q\left(r_{A,c}^{t}\right)=\sqrt{r_{A,c}^{t}}$.

Alice’s payoff is defined as the overall satisfaction received by the selected IoT nodes’ invested effort while considering her personal cost to provide the corresponding rewards to the selected nodes, and it is formulated as follows

(5) $$U_{A}^{t}\left(e\right)=\sum_{c=1}^{|C_{A}^{t}|}\left[\rho_{c}^{t}\left(e_{A,c}^{t}-\lambda \cdot r_{A,c}^{t}\right)\right]$$

where $\lambda \in R^{+}$ denotes Alice’s cost to provide rewards, and $e=[e_{A,1}^{t},\;\ldots ,\;e_{A,|C_{A}^{t}|}^{t}]$ is the nodes’ effort vector. In a scenario where Alice is unaware of the selected nodes’ scores and their potential to provide effort in the crowdsourcing process, Alice probabilistically estimates each node’s score with probability $\rho_{c}^{t}$, where $\sum_{c=1}^{|C_{A}^{t}|}\rho_{c}^{t}=1$. We exploit the Bayesian trust belief $\mu_{A,c}^{t}$ to determine the probability $\rho_{c}^{t}$, as follows.

(6) $$\rho_{c}^{t}=\frac{\mu_{A,c}^{t}}{\sum_{c=1}^{C_{A}^{t}}\mu_{A,c}^{t}}$$

Based on Equations (4) and (5), we define the social welfare as the net gain of all participants in the process.

(7) $$SW\left(e\right)=U_{A}^{t}\left(e\right)+\sum_{c=1}^{|C_{A}^{t}|}U_{c}^{t}\left(e_{A,c}^{t}\right).$$

## 3. Contract-Theoretic Crowdsourcing

The interactions among Alice and the selected IoT nodes are captured via a contract-theoretic trust-based crowdsourcing model aiming at determining the optimal contracts that facilitate the crowdsourcing process. Initially, the complete information scenario regarding the nodes’ scores (i.e., trustworthiness) is considered for benchmarking purposes. Then, the realistic scenario of incomplete information is presented, where Alice probabilistically estimates the nodes’ scores based on the probability $\rho_{c}^{t}$. The probability distribution is updated, while Alice interacts with the nodes.

Based on the proposed contract-theoretic model [21] Alice can deal with the information incompleteness and efficiently incentivize the selected nodes to contribute to the crowdsourcing process. Specifically, an optimization problem is solved by Alice (see Section 3.3), where she determines the optimal contracts $\left\{e_{A,c}^{t*},r_{A,c}^{t*}\right\}$ towards her overall satisfaction (Equation (5)) as well as the selected IoT nodes’ (Equation (4)) payoff joint maximization. Thus, the contract-theoretic efforts of the IoT nodes are estimated based on the rewards provided by Alice, the reported trustworthiness scores $p_{c}^{t}$, and the probability distribution $\rho_{c}^{t}$ in order for their perceived payoff to be maximized. In the following analysis, we assume that Alice has already selected the IoT nodes $C_{A}^{t}$ that will participate in the crowdsourcing process, while the detailed analysis of nodes’ selection based on the theory of Stochastic Learning Automata (SLA) is shown in Section 4.

### 3.1. Complete Information Scenario

In this section, we examine the ideal benchmarking scenario, where Alice has complete information of the selected IoT nodes’ trustworthiness scores, i.e., $p_{c}^{t},\forall c\in C$ is known. Alice can fully exploit the nodes’ invested efforts and maximize her payoff while guaranteeing that their achieved benefits (Equation (4)) are optimized. The condition of individual rationality should hold true in the offered contract such that the nodes are incentivized to participate in the crowdsourcing.

**Definition** **1.** *(Individual Rationality (IR)) A contract $\left\{e_{A,c}^{t},r_{A,c}^{t}\right\}$ satisfies the IR condition if every node experiences a non-negative payoff, i.e., $U_{c}^{t}\left(e_{A,c}^{t}\right)\geq 0,\forall c\in C_{A}^{t}$.*

The following optimization problem is introduced to determine the optimal contracts among Alice and each selected node.

(8) $$\max_{{\left\{e_{A,c}^{t},r_{A,c}^{t}\right\}}_{\forall c\in C_{A}^{t}}}\left[e_{A,c}^{t}-\lambda \cdot r_{A,c}^{t}\right]$$

(9) $$s.t.\;p_{c}^{t}\cdot q\left(r_{A,c}^{t}\right)-e_{A,c}^{t}\geq 0,\forall c\in C_{A}^{t}$$

Alice aims at maximizing her payoff by providing the minimum acceptable payoff to each IoT node *c*, $c\in C_{A}^{t}$. Thus, As a result, the constraint (9) is reduced to an equality as follows.

(10) $$p_{c}^{t}\cdot q\left(r_{A,c}^{t}\right)-e_{A,c}^{t}=0,\forall c\in C_{A}^{t}$$

**Theorem** **2.** *In the complete information scenario, the optimal contract among Alice and each IoT node c, $c\in C_{A}^{t}$ is $\left\{e_{A,c}^{t*},r_{A,c}^{t*}\right\}=\left\{\frac{{\left(p_{c}^{t}\right)}^{2}}{2\lambda },{\left(\frac{p_{c}^{t}}{2\lambda }\right)}^{2}\right\}$.*

**Proof** **of** **Theorem** **2.** Based on the reduced constraint in Equation (10) we have:

(11) $$\begin{array}{cc} \; & p_{c}^{t}\cdot \sqrt{r_{A,c}^{t}}-e_{A,c}^{t}=0\\ \; & \overset{r_{A,c}^{t}\in \left[0,1\right]}{⟷}r_{A,c}^{t}={\left(\frac{e_{A,c}^{t}}{p_{c}^{t}}\right)}^{2} \end{array}$$

Thus, from Equation (5) we have that the following holds true:

(12) $$U_{A,c}^{t}=e_{A,c}^{t}-\lambda \cdot {\left(\frac{e_{A,c}^{t}}{p_{c}^{t}}\right)}^{2}$$

As a result, in order to find the optimal contract that Alice offers to each IoT device, we consider the first order derivative of $U_{A,c}^{t}$ with respect to the effort $e_{A,c}^{t}$ and we set it equal to 0, as follows.

(13) $$\begin{array}{cc} \; & \frac{\partial U_{A,c}^{t}}{\partial (e_{A,c}^{t})}=0\\ \; & ⟺1-2\lambda \frac{e_{A,c}^{t}}{{\left(p_{c}^{t}\right)}^{2}}=0\\ \; & ⟺e_{A,c}^{t}=\frac{{\left(p_{c}^{t}\right)}^{2}}{2\lambda } \end{array}$$

Based on Equations (11) and (13) we have that the following holds true:

(14) $$r_{A,c}^{t}={\left(\frac{p_{c}^{t}}{2\lambda }\right)}^{2}$$

Thus, the optimal contract under the complete information setting is given by $\left\{e_{A,c}^{t},r_{A,c}^{t}\right\}=\left\{\frac{{\left(p_{c}^{t}\right)}^{2}}{2\lambda },{\left(\frac{p_{c}^{t}}{2\lambda }\right)}^{2}\right\}$. □

### 3.2. Feasible Contract under Incomplete Information

In this section, we study the realistic scenario of incomplete information regarding the IoT nodes’ scores. In a real-life IoT crowdsourcing scenario, the IoT nodes may not reveal their level of trustworthiness, or even worse, they may maliciously advertise fake information regarding their scores. Thus, Alice probabilistically estimates the IoT nodes’ scores by interacting with them over time and updating her probability $\rho_{c}^{t}$ regarding each node’s *c* score via updating her posterior trust belief $\mu_{A,c}^{t}$. Alice aims to maximize her benefit (Equation (5)) by interacting with the selected nodes, while guaranteeing their payoff maximization (Equation (4)) via determining the optimal contract $\left\{e_{A,c}^{t*},r_{A,c}^{t*}\right\}$. To determine the optimal efforts and rewards, the criteria of individual rationality (IR), incentive compatibility (IC), fairness, monotonicity, and rationality should hold true, as analyzed below.

**Definition** **2.** *(Incentive Compatibility (IC)) Each IoT node must select the contract $\left\{e_{A,c}^{t},r_{A,c}^{t}\right\}$ designed for its own score $p_{c}^{t}$, i.e., $p_{c}^{t}\cdot q\left(r_{A,c}^{t}\right)-e_{A,c}^{t}\geq p_{c}^{t}\cdot q\left(r_{A,c^{\prime }}^{t}\right)-e_{A,c^{\prime }}^{t},\forall c,c^{\prime }\in C_{A}^{t},c\neq c^{\prime }$.*

The physical meaning of the IC condition is that each node should select its personalized contract in order to optimize its benefit from participating in crowdsourcing.

**Proposition** **1.** *(Fairness) A contract must be fair: $r_{A,c}^{t}>r_{A,c^{\prime }}^{t}\Leftrightarrow p_{c}^{t}>p_{c^{\prime }}^{t}$, $r_{A,c}^{t}=r_{A,c^{\prime }}^{t}\Leftrightarrow p_{c}^{t}=p_{c^{\prime }}^{t},\forall c\neq c^{\prime }\in C_{A}^{t}$.*

**Proof** **of** **Proposition** **1.** We prove that $p_{c}^{t}>p_{c^{\prime }}^{t}\Rightarrow r_{A,c}^{t}>r_{A,c^{\prime }}^{t}$, by utilizing the IC condition: $p_{c}^{t}\cdot q\left(r_{A,c}^{t}\right)-e_{A,c}^{t}\geq p_{c}^{t}\cdot q\left(r_{A,c^{\prime }}^{t}\right)-e_{A,c^{\prime }}^{t}$ and $p_{c^{\prime }}^{t}\cdot q\left(r_{A,c^{\prime }}^{t}\right)-e_{A,c^{\prime }}^{t}\geq p_{c^{\prime }}^{t}\cdot q\left(r_{A,c}^{t}\right)-e_{A,c}^{t}$. By adding those inequalities, we have: $p_{c}^{t}\cdot q\left(r_{A,c}^{t}\right)+p_{c^{\prime }}^{t}\cdot q\left(r_{A,c^{\prime }}^{t}\right)\geq p_{c}^{t}\cdot q\left(r_{A,c^{\prime }}^{t}\right)+p_{c^{\prime }}^{t}\cdot q\left(r_{A,c}^{t}\right)\Leftrightarrow \left(p_{c}^{t}-p_{c^{\prime }}^{t}\right)\cdot \left[q\left(r_{A,c}^{t}\right)-q\left(r_{A,c^{\prime }}^{t}\right)\right]\geq 0$. Given that $p_{c}^{t}>p_{c^{\prime }}^{t}$ and $q(r_{A,c}^{t})$ is a strictly increasing function with respect to $r_{A,c}^{t}$, we conclude that $r_{A,c}^{t}>r_{A,c^{\prime }}^{t}$. Then, we prove that $r_{A,c}^{t}>r_{A,c^{\prime }}^{t}\Rightarrow p_{c}^{t}>p_{c^{\prime }}^{t}$. It holds true that $r_{A,c}^{t}>r_{A,c^{\prime }}^{t}$ and $q(r_{A,c}^{t})$ is a strictly increasing function with respect to $r_{A,c}^{t}$, thus, $q\left(r_{A,c}^{t}\right)-q\left(r_{A,c^{\prime }}^{t}\right)>0$. Thus, from $\left(p_{c}^{t}-p_{c^{\prime }}^{t}\right)\cdot \left[q\left(r_{A,c}^{t}\right)-q\left(r_{A,c^{\prime }}^{t}\right)\right]\geq 0$, we conclude that $p_{c}^{t}>p_{c^{\prime }}^{t}$. Similarly, we can also show that $r_{A,c}^{t}=r_{A,c^{\prime }}^{t}\Leftrightarrow p_{c}^{t}=p_{c^{\prime }}^{t}$. □

The physical meaning of Proposition 1 is that a contract should be fair in order to incentivize the nodes to participate in the crowdsourcing by providing higher rewards to the IoT nodes of higher scores, which have the potential to contribute more in the crowdsourcing process.

**Proposition** **2.** *(Monotonicity) An IoT node of higher score, i.e., $p_{1}^{t}<\cdots <p_{c}^{t}<\cdots <p_{|C_{A}^{t}|}^{t}$, will receive a greater reward, i.e., $r_{A,1}^{t}<\cdots <r_{A,c}^{t}<\cdots <r_{A,|C_{A}^{t}|}^{t}$ by providing a higher effort, i.e., $e_{A,1}^{t}<\cdots <e_{A,c}^{t}<\cdots <e_{A,|C_{A}^{t}|}^{t}$.*

**Proof** **of** **Proposition** **2.** We have sorted the IoT nodes as $p_{1}^{t}<\cdots <p_{c}^{t}<\cdots <p_{|C_{A}^{t}|}^{t}$. Thus, the first part of the proof stems from Proposition 1. Based on the IC condition for $p_{c}^{t}>p_{c^{\prime }}^{t},\forall c\neq c^{\prime }\in C_{A}^{t}$ and assuming $e_{A,c}^{t}>e_{A,c^{\prime }}^{t}$, we have that $p_{c}^{t}\cdot q\left(r_{A,c}^{t}\right)-e_{A,c}^{t}\geq p_{c}^{t}\cdot q\left(r_{A,c^{\prime }}^{t}\right)-e_{A,c^{\prime }}^{t}\Leftrightarrow p_{c}^{t}\cdot \left(q\left(r_{A,c}^{t}\right)-q\left(r_{A,c^{\prime }}^{t}\right)\right)\geq e_{A,c}^{t}-e_{A,c^{\prime }}^{t}$. Given that $q(r_{A,c}^{t})$ is a strictly increasing function with respect to $r_{A,c}^{t}$, we conclude that $r_{A,c}^{t}>r_{A,c^{\prime }}^{t}$. Then, assuming that $r_{A,c}^{t}>r_{A,c^{\prime }}^{t}$ and based on the IC condition, we have that $p_{c^{\prime }}^{t}\cdot q\left(r_{A,c^{\prime }}^{t}\right)-e_{A,c^{\prime }}^{t}\geq p_{c^{\prime }}^{t}\cdot q\left(r_{A,c}^{t}\right)-e_{A,c}^{t}\Leftrightarrow e_{A,c}^{t}-e_{A,c^{\prime }}^{t}\geq p_{c^{\prime }}^{t}\cdot \left(q\left(r_{A,c}^{t}\right)-q\left(r_{A,c^{\prime }}^{t}\right)\right)$. Since $r_{A,c}^{t}>r_{A,c^{\prime }}^{t}$ and given that $q(r_{A,c}^{t})$ is a strictly increasing function with respect to $r_{A,c}^{t}$, we conclude that $e_{A,c}^{t}>e_{A,c^{\prime }}^{t}$. □

The physical meaning of the monotonicity condition is that a node of a higher score, i.e., trustworthiness, should receive a higher reward, as it will eventually invest a higher effort.

In the following proposition, we analyze the perceived payoff of devices that are characterized by different scores.

**Proposition** **3.** *(Rationality) An IoT node of a higher score, i.e., $p_{1}^{t}<\cdots <p_{c}^{t}<\cdots <p_{|C_{A}^{t}|}^{t}$, will experience a higher payoff, i.e., $U_{1}^{t}\left(e_{A,1}^{t}\right)<\cdots <U_{c}^{t}\left(e_{A,c}^{t}\right)<\cdots <U_{|C_{A}^{t}|}^{t}\left(e_{A,|C_{A}^{t}|}^{t}\right)$.*

**Proof** **of** **Proposition** **3.** We examine two indicative nodes *c*, $c^{\prime }\in C_{A}^{t},c\neq c^{\prime }$, with with $p_{c}^{t}>p_{c^{\prime }}^{t}$. By utilizing the IC condition, we have $p_{c}^{t}\cdot q\left(r_{A,c}^{t}\right)-e_{A,c}^{t}\geq p_{c}^{t}\cdot q\left(r_{A,c^{\prime }}^{t}\right)-e_{A,c^{\prime }}^{t}\geq p_{c^{\prime }}^{t}\cdot q\left(r_{A,c^{\prime }}^{t}\right)-e_{A,c^{\prime }}^{t}$. Thus, $U_{c}^{t}\left(e_{A,c}^{t}\right)>U_{c^{\prime }}^{t}\left(e_{A,c^{\prime }}^{t}\right)$. □

The physical meaning of the rationality condition is that an IoT node of a higher score, given that it invests greater effort in the crowdsourcing process by receiving a greater reward, will ultimately achieve a greater payoff.

Following the above analysis, our goal is to determine the optimal contract between Alice and each selected IoT node aiming at maximizing Alice’s achieved payoff and jointly optimizing each IoT node’s payoff, while accounting for the incomplete information. The corresponding optimization problem is defined as follows

(15a) $$P1:\max_{{\left(e_{A,c}^{t},r_{A,c}^{t}\right)}_{\forall c\in C_{A}^{t}}}U_{A}^{t}\left(e\right)=\sum_{c=1}^{|C_{A}^{t}|}\left[\rho_{c}^{t}\left(e_{A,c}^{t}-\lambda \cdot r_{A,c}^{t}\right)\right]$$

(15b) $$s.t.\;p_{c}^{t}\cdot q\left(r_{A,c}^{t}\right)-e_{A,c}^{t}\geq 0,\forall c\in C_{A}^{t}$$

(15c) $$p_{c}^{t}\cdot q\left(r_{A,c}^{t}\right)-e_{A,c}^{t}\geq p_{c}^{t}\cdot q\left(r_{A,c^{\prime }}^{t}\right)-e_{A,c^{\prime }}^{t},\forall c\neq c^{\prime }\in C_{A}^{t}$$

(15d) $$0\leq r_{A,1}^{t}<\cdots <r_{A,c}^{t}<\cdots <r_{A,|C_{A}^{t}|}^{t}$$

where Equations (15b) and (15c) capture the IR and IC conditions, respectively, and Equation (15d) jointly represents the fairness, monotonicity, and rationality conditions. The optimization problem **P1** is non-convex. In the following section, we present an analysis to reduce its constraints and determine its solution.

### 3.3. Optimal Contract under Incomplete Information

Towards solving the optimization problem (15a)–(15d), initially, we reduce the IR constraint in Equation (15b). Given that $p_{1}^{t}<\cdots <p_{c}^{t}<\cdots <p_{|C_{A}^{t}|}^{t}$ and based on the IC condition, we have $p_{c}^{t}\cdot q\left(r_{A,c}^{t}\right)-e_{A,c}^{t}\geq p_{c}^{t}\cdot q\left(r_{A,c^{\prime }}^{t}\right)-e_{A,c^{\prime }}^{t}\geq p_{c}^{t}\cdot q\left(r_{A,1}^{t}\right)-e_{A,1}^{t}\geq^{\left(IR\right)}0$. Thus, if the IR constraint of the IoT node with the lowest score $p_{1}^{t}$ is satisfied, then $p_{c}^{t}\cdot q\left(r_{A,c}^{t}\right)-e_{A,c}^{t}\geq 0$ holds true for each IoT node $c\in C_{A}^{t}$. Given that Alice will try to exploit the maximum benefit from the nodes’ invested effort, the reduced IR constraint can be further reduced to equality, i.e., $p_{1}^{t}\cdot q\left(r_{A,1}^{t}\right)-e_{A,1}^{t}=0$.

Focusing on the reduction of the IC constraints in Equation (15c), we introduce the following terminology: (a) Downward IC (DIC) constraints between the nodes *c*, $c^{\prime }$, $c^{\prime }\in \left\{1,\;\ldots ,\;c-1\right\}$, (b) Upward IC (UIC) constraints between the nodes *c*, $c^{\prime }$, $c^{\prime }\in \{c+1,\;\ldots ,\;|C_{A}^{t}\left|\right\}$, (c) Local Downward IC (LDIC) constraints between the adjacent nodes *c*, $c-1\in C_{A}^{t}$, and (d) Local Upward IC (LUIC) constraints between the adjacent nodes *c*, $c+1\in C_{A}^{t}$.

**Proposition** **4.** *All the DIC constraints can be represented by the LDIC constraints.*

**Proof** **of** **Proposition** **4.** We consider three adjacent scores of nodes, i.e., $p_{c-1}^{t}<p_{c}^{t}<p_{c+1}^{t}$ and we can write the IC constraints as: $p_{c+1}^{t}\cdot q\left(r_{A,c+1}^{t}\right)-e_{A,c+1}^{t}\geq p_{c+1}^{t}\cdot q\left(r_{A,c}^{t}\right)-e_{A,c}^{t}$ and $p_{c}^{t}\cdot q\left(r_{A,c}^{t}\right)-e_{A,c}^{t}\geq p_{c}^{t}\cdot q\left(r_{A,c-1}^{t}\right)-e_{A,c-1}^{t}$. We know that $r_{A,c}^{t}>r_{A,c-1}^{t}\overset{q↗}{⟺}q\left(r_{A,c}^{t}\right)-q\left(r_{A,c-1}^{t}\right)>0$. Thus, for $p_{c+1}^{t}>p_{c}^{t}$, we have $p_{c+1}^{t}\cdot \left[q\left(r_{A,c}^{t}\right)-q\left(r_{A,c-1}^{t}\right)\right]>p_{c}^{t}\cdot \left[q\left(r_{A,c}^{t}\right)-q\left(r_{A,c-1}^{t}\right)\right]$. Given that $p_{c}^{t}\cdot q\left(r_{A,c}^{t}\right)-e_{A,c}^{t}\geq p_{c}^{t}\cdot q\left(r_{A,c-1}^{t}\right)-e_{A,c-1}^{t}$, we conclude that $p_{c+1}^{t}\cdot \left[q\left(r_{A,c}^{t}\right)-q\left(r_{A,c-1}^{t}\right)\right]>p_{c}^{t}\cdot \left[q\left(r_{A,c}^{t}\right)-q\left(r_{A,c-1}^{t}\right)\right]\geq e_{A,c}^{t}-e_{A,c-1}^{t}$. By recursively applying the latter outcome, we have that $p_{c+1}^{t}\cdot q\left(r_{A,c+1}^{t}\right)-e_{A,c+1}^{t}\geq p_{c+1}^{t}\cdot q\left(r_{A,c}^{t}\right)-e_{A,c}^{t}\geq p_{c+1}^{t}\cdot q\left(r_{A,c-1}^{t}\right)-e_{A,c-1}^{t}\geq \cdots \geq p_{c+1}^{t}\cdot q\left(r_{A,1}^{t}\right)-e_{A,1}^{t}$. Thus, all the DIC constraints can be equivalently captured by the LDIC constraint $p_{c}^{t}\cdot q\left(r_{A,c}^{t}\right)-e_{A,c}^{t}\geq p_{c}^{t}\cdot q\left(r_{A,c-1}^{t}\right)-e_{A,c-1}^{t}$. □

**Proposition** **5.** *All the UIC constraints can be represented by the LDIC constraints.*

**Proof** **of** **Proposition** **5.** We consider three adjacent scores of nodes, i.e., $p_{c-1}^{t}<p_{c}^{t}<p_{c+1}^{t}$ and we write the IC constraints as: $p_{c-1}^{t}\cdot q\left(r_{A,c-1}^{t}\right)-e_{A,c-1}^{t}\geq p_{c-1}^{t}\cdot q\left(r_{A,c}^{t}\right)-e_{A,c}^{t}$ and $p_{c}^{t}\cdot q\left(r_{A,c}^{t}\right)-e_{A,c}^{t}\geq p_{c}^{t}\cdot q\left(r_{A,c+1}^{t}\right)-e_{A,c+1}^{t}$. From Proposition 1, we have $r_{A,c}^{t}>r_{A,c-1}^{t}\Leftrightarrow p_{c}^{t}>p_{c-1}^{t}$, thus, from the latter inequality we derive: $e_{A,c+1}^{t}-e_{A,c}^{t}\geq p_{c}^{t}\cdot \left[q\left(r_{A,c+1}^{t}\right)-q\left(r_{A,c}^{t}\right)\right]\geq p_{c-1}^{t}\cdot \left[q\left(r_{A,c+1}^{t}\right)-q\left(r_{A,c}^{t}\right)\right]$. Based on the latter outcome, we have: $p_{c-1}^{t}\cdot q\left(r_{A,c-1}^{t}\right)-e_{A,c-1}^{t}\geq p_{c-1}^{t}\cdot q\left(r_{A,c}^{t}\right)-e_{A,c}^{t}\geq p_{c-1}^{t}\cdot q\left(r_{A,c+1}^{t}\right)-e_{A,c+1}^{t}\geq \cdots \geq p_{c-1}^{t}\cdot q\left(r_{A,|C_{A}^{t}|}^{t}\right)-e_{A,|C_{A}^{t}|}^{t}$. □

Based on Propositions 4 and 5, we observe that the $|C_{A}^{t}\left|\cdot (\right|C_{A}^{t}\left|-1\right)$ IC constraints defined in the original optimization problem P1 are efficiently reduced to $|C_{A}^{t}|-1$ constraints.

Based on the reduced IR and IC constraints, the optimization problem **P1** can be rewritten as follows.

(16a) $$P2:\max_{{\left(e_{A,c}^{t},r_{A,c}^{t}\right)}_{\forall c\in C_{A}^{t}}}U_{A}^{t}\left(e\right)=\sum_{c=1}^{|C_{A}^{t}|}\left[\rho_{c}^{t}\left(e_{A,c}^{t}-\lambda \cdot r_{A,c}^{t}\right)\right]$$

(16b) $$s.t.\;p_{1}^{t}\cdot q\left(r_{A,1}^{t}\right)-e_{A,1}^{t}=0$$

(16c) $$p_{c}^{t}\cdot q\left(r_{A,c}^{t}\right)-e_{A,c}^{t}\geq p_{c}^{t}\cdot q\left(r_{A,c-1}^{t}\right)-e_{A,c-1}^{t},\forall c\in C_{A}^{t}$$

(16d) $$0\leq r_{A,1}^{t}<\cdots <r_{A,c}^{t}<\cdots <r_{A,|C_{A}^{t}|}^{t}$$

The optimization problem **P2** is convex and can be solved with standard optimization tools to determine the optimal nodes’ effort vector $e^{*}=\left[e_{A,1}^{*t},\ldots ,e_{A,c}^{*t},\ldots ,e_{A,C_{A}^{t}}^{*t}\right]$ and Alice’s reward vector $r^{*}=\left[r_{A,1}^{*t},\ldots ,r_{A,c}^{*t},\ldots ,r_{A,C_{A}^{t}}^{*t}\right]$.

## 4. Autonomous Reinforcement Learning-Based Contributors Selection

In this section, we propose a distributed reinforcement learning (RL) model based on the theory of Stochastic Learning Automata (SLA) that enables Alice to select $|C_{A}^{t}|$ nodes to facilitate her interaction with Bob. Alice’s discrete action space consists of vectors $A_{s}^{t}=\left[c,c^{\prime },\ldots ,c_{|C_{A}^{t}|}\right]$, where $c,c^{\prime },c_{|C_{A}^{t}|}\in C$, and $s\in S^{t}=\left\{1,\ldots ,s,\ldots ,\right|S^{t}\left|\right\}$ where $|S^{t}|$ is the total number of subsets of the |C| nodes with cardinality $|C_{A}^{t}|$ [22]. Alice aims at minimizing her communication delay with the selected nodes, thus, she prefers to select nodes with small physical distance and low congestion $cr_{c}^{t}$ [23,24]. Moreover, she considers the nodes’ scores, as they are reported by them by transmitting respective wireless beacons (Section 2.3), while also weighing the reported values based on her probabilistic trust belief. This process leads Alice to formulate the relative scores of the IoT nodes. Thus, Alice determines the personalized feedback $F_{A,A_{s}^{t}}^{\left(ite,t\right)}$ at the ite iteration of the SLA algorithm at time slot *t* by choosing the action vector $A_{s}^{t}$ as:

(17) $$F_{A,A_{s}^{t}}^{\left(ite,t\right)}=\sum_{c\in A_{s}^{t}}\left[\frac{p_{c}^{t}\cdot \rho_{c}^{t}}{cr_{c}^{t}\cdot \frac{d_{c}^{t}}{\sum_{c\in C}d_{c}^{t}}}\right].$$

**Figure 1:**
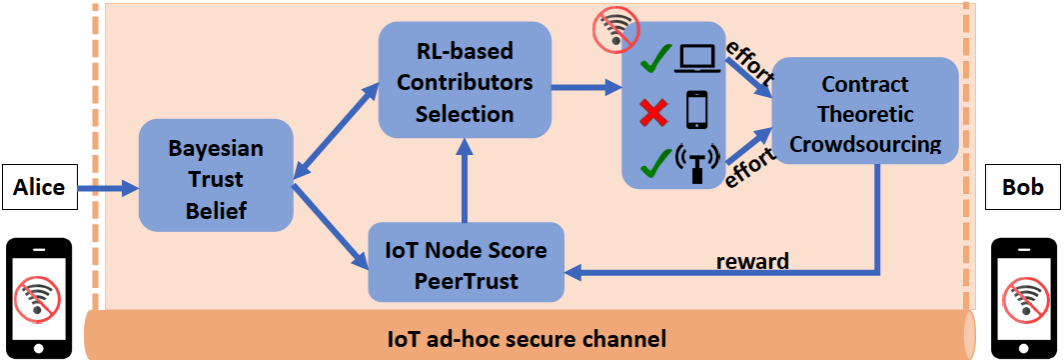
General Architecture.

The personalized feedback is engineered in such a way that enables Alice to act as an autonomous RL agent within the IoT network. Specifically, if $F_{A,A_{s}^{t}}^{\left(ite,t\right)}$ is high then her action $A_{s}^{t}$ at the iteration ite of time instance *t* is good since the chosen subset $s\in S^{t}$ of the IoT nodes is characterized by a good cumulative trust profile and satisfactory cumulative network characteristics. Thus, Alice chooses trustworthy IoT nodes for the crowdsourcing process in order for the interaction with Bob to be secure and successful, and at the same time not further congest the IoT network. $F_{A,A_{s}^{t}}^{\left(ite,t\right)}$ in Equation (17) is normalized as ${\hat{F}}_{A,A_{s}^{t}}^{\left(ite,t\right)}=\sum_{c\in A_{s}^{t}}\left[\frac{p_{c}^{t}\cdot \rho_{c}^{t}}{cr_{c}^{t}\cdot \frac{d_{c}^{t}}{\sum_{c\in C}d_{c}^{t}}}\right]/\sum_{c\in C}\left[\frac{p_{c}^{t}\cdot \rho_{c}^{t}}{cr_{c}^{t}\cdot \frac{d_{c}^{t}}{\sum_{c\in C}d_{c}^{t}}}\right]$, thus, $0\leq {\hat{F}}_{A,A_{s}^{t}}^{\left(ite,t\right)}\leq 1,\forall ite,t$. Given the personalized feedback, Alice determines her action probability vector ${\mathrm{Pr}}_{A}^{\left(\mathrm{ite},t\right)}=\left[Pr_{A,1}^{\left(ite,t\right)},\ldots ,Pr_{A,A_{s}^{t}}^{\left(ite,t\right)},\ldots ,Pr_{A,A_{\left|S\right|}^{t}}^{\left(ite,t\right)}\right]$, which is updated based on the SLA gradient ascent rule as follows:

(18a) $$Pr_{A,A_{\left|S\right|}^{t}}^{\left(ite+1,t\right)}=Pr_{A,A_{\left|S\right|}^{t}}^{\left(ite,t\right)}+b{\hat{F}}_{A,A_{s}^{t}}^{\left(ite,t\right)}\left(1-Pr_{A,A_{\left|S\right|}^{t}}^{\left(ite,t\right)}\right),\overset{\left(ite+1,t\right)}{A_{\left|S\right|}}=\overset{\left(ite,t\right)}{A_{\left|S\right|}}$$

(18b) $$Pr_{A,A_{\left|S\right|}^{t}}^{\left(ite+1,t\right)}=Pr_{A,A_{\left|S\right|}^{t}}^{\left(ite,t\right)}-b{\hat{F}}_{A,A_{s}^{t}}^{\left(ite,t\right)}Pr_{A,A_{\left|S\right|}^{t}}^{\left(ite,t\right)},\overset{\left(ite+1,t\right)}{A_{\left|S\right|}}\neq \overset{\left(ite,t\right)}{A_{\left|S\right|}}$$

where 0<b≤1 is the learning parameter. For higher values of *b*, Alice explores less her action space, which may lead her to inefficient but faster decisions. Equation (18a) expresses the probability of Alice selecting the same action $A_{\left|S\right|}^{t}$ in iteration ite, while Equation (18b) depicts the probability of choosing a different action. It is noted that at ite=0, Alice selects an action with equal probability. The SLA algorithm enables Alice to converge to the optimal selection of $|C_{A}^{t}|$ nodes in an iterative manner. In Figure 1, we present the overall architecture of the proposed model.

## 5. Numerical Results

In this section, we provide a detailed performance evaluation of the proposed offline contract-theoretic crowdsourcing framework via modeling and simulation. The operation of the stochastic learning automata-based nodes’ selection by Alice and the evaluation of the introduced Bayesian trust belief model, are presented in Section 5.1. A thorough evaluation of the proposed offline contract-theoretic crowdsourcing framework is discussed in Section 5.2, and a comparative analysis is provided in Section 5.3.

The simulation parameters’ values are presented in Table 2, unless otherwise explicitly stated. The proposed framework’s evaluation was conducted in a MacBook Pro Laptop, 2.5 GHz Intel Core i7 with 16 GB LPDDR3 available RAM.

### 5.1. Stochastic Learning Automata Operation & Bayesian Trust Belief Evaluation

**Figure 2:**
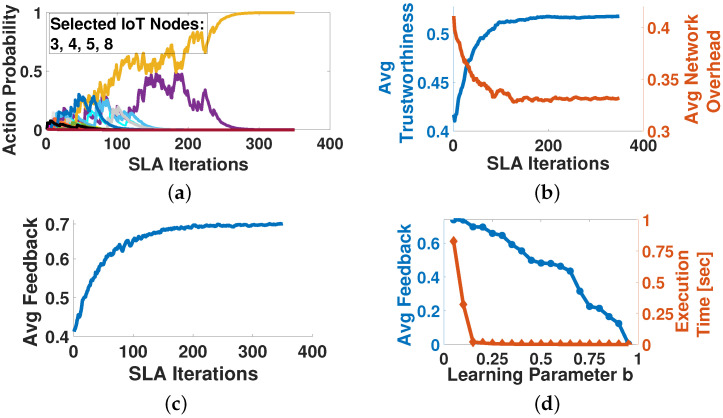
Stochastic Learning Automata operation and performance evaluation. (**a**) Action Probability vs. Iterations, (**b**) Average Trustworthiness & Network Overhead vs. Iterations, (**c**) Average Personalized Feedback vs. Iterations, (**d**) Average Personalized Feedback and Convergence Time vs. *b*.

Figure 2 illustrates the performance characteristics of the SLA algorithm. Each data point represents an aggregate of multiple simulations where congestion values for each selected IoT node are generated in a Monte Carlo fashion. Figure 2a indicates the convergence of Alice’s action probabilities to the set of $|C_{A}^{t}|$ nodes that will participate in the crowdsourcing process at a specific time instance. Specifically, the probability of selecting the IoT nodes 3, 4, 5, 8 converges to 1, while the probabilities of selecting any other subset of IoT nodes, i.e., a different IoT nodes combination of cardinality $|C_{A}^{t}|$, converges to 0. The results reveal that the SLA algorithm converges fast (less than 400 SLA iterations, equivalent to 0.8 s).

Figure 2b presents the convergence of Alice’s achieved average trustworthiness $T_{c}$ and network overhead with respect to the number of selected nodes, where the latter is captured as $d_{c}^{t}\cdot cr_{c}^{t}$. The results reveal that Alice, by acting as a Stochastic Learning Automaton, is able to autonomously select nodes that are characterized by high trustworthiness, while simultaneously possessing a low congestion rate, resulting in low average network overhead. Thus, Alice receives a high personalized feedback ${\hat{F}}_{A,A_{s}^{t}}^{\left(ite,t\right)}$ (Figure 2c). Figure 2d, presents the convergence time and the corresponding average received personalized feedback as a function of the learning parameter *b*. The results show that for increasing values of *b*, the convergence efficiency increases, however, the average received personalized feedback for the selected action decreases, due to under-exploration of the action space leading to sub-optimal exploitation of resources.

**Figure 3:**
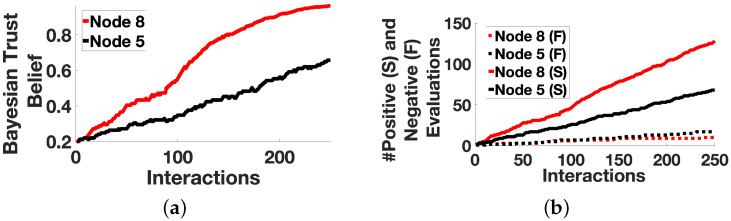
Bayesian trust belief evaluation (*S*: positive, *F*: negative evaluations). (**a**) Trust Belief vs. Interactions, (**b**) Evaluations vs. Interactions.

In Figure 3, we study the operation of the proposed framework in terms of modeling the Bayesian trust belief $\mu_{A,c}^{t}$ for two indicative nodes with IDs 8 and 5 throughout the time horizon (i.e., for 250 examined interactions). Figure 3a shows that Alice has obtained higher Bayesian trust belief for IoT node 8, given that this node receives a higher number of positive evaluations, i.e., $S_{8}^{t}>S_{5}^{t},\forall t$, and a lower number of negative evaluations over the examined time horizon (Figure 3b). Thus, as proven in Theorem 1, Alice trusts IoT node 8 more for her interactions within the offline environment.

### 5.2. Contract-Theoretic Crowdsourcing Evaluation

**Figure 4:**
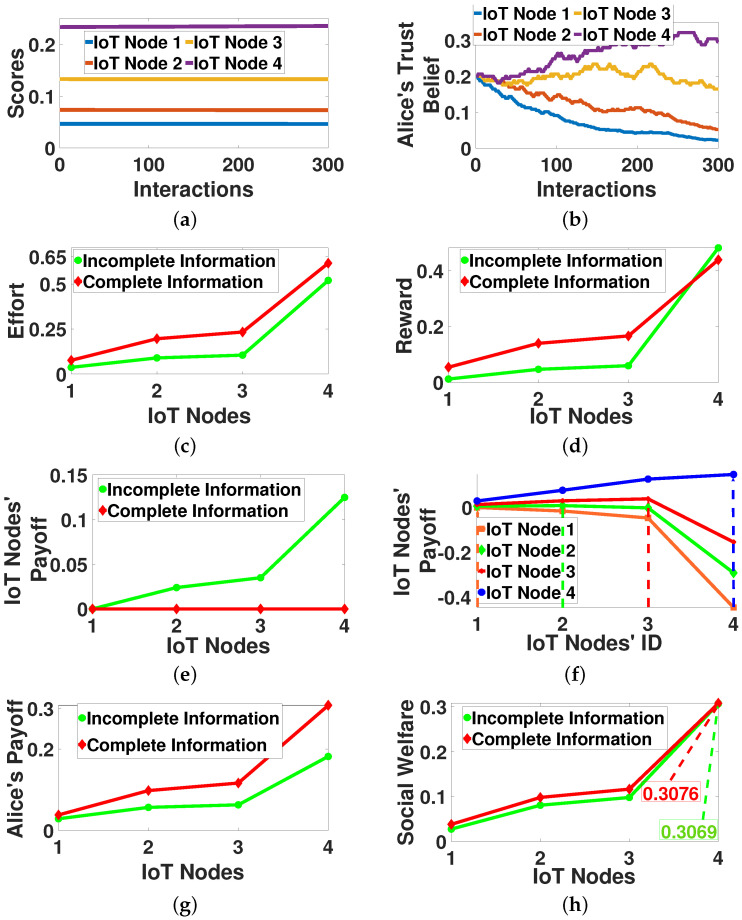
Offline contract-theoretic crowdsourcing—operation and performance evaluation. (**a**) Nodes’ Scores vs. Interactions, (**b**) Alice’s Belief vs. Interactions, (**c**) Effort vs. Nodes, (**d**) Reward vs. Nodes, (**e**) Nodes’ Payoff vs. Nodes, (**f**) Nodes’ Payoff vs. Nodes IDs, (**g**) Alice’s Payoff vs. Nodes, (**h**) Social Welfare vs. Nodes.

In this section, we evaluate the contract-theoretic crowdsourcing model, considering an indicative time slot t=300, where Alice has already selected the set of nodes $C_{A}^{300}$ that will participate in the crowdsourcing process. Figure 4a,b present the evolution of the nodes’ scores and Alice’s Bayesian trust belief throughout the time horizon of 200 interactions. Regarding the latter ones, different distributions have been adopted for the values of $S_{c}^{t}$ and $F_{c}^{t}$ to capture the stochasticity of the IoT nodes’ contribution to the crowdsourcing.

The results reveal that Alice gains knowledge from her interactions with the nodes and builds a Bayesian trust belief that follows the same trend as the nodes’ scores, even if she operates under the incomplete information scenario. Figure 4c–e show the nodes’ efforts, their provided rewards by Alice, and their payoffs under both the complete and incomplete information scenarios. Figure 4f demonstrates the nodes’ payoff by receiving any contract designed for each IoT node. Figure 4g,h illustrates Alice’s payoff and the overall examined system’s social welfare (Equation (7)) in an aggregated manner at t=300.

The results reveal that when Alice is fully aware of the nodes’ scores (complete information), she fully exploits the nodes’ efforts (Figure 4c), by providing high rewards to them (Figure 4d). Thus, she experiences high payoffs (Figure 4g). Figure 4e shows that the experienced payoff by each node is equal to 0, as Alice knows the nodes’ exact scores and the rewards are enough to optimally satisfy their IR conditions (Definition 1). Focusing on the incomplete information scenario, the nodes experience a higher payoff (Figure 4e) given that Alice cannot precisely predict their scores.

Correspondingly, Alice experiences a lower payoff compared to the complete information scenario (Figure 4g). Based on Figure 4f, we observe that the nodes achieve their highest personal payoff under the incomplete information scenario, only when they are offered a personalized contract aligned with their scores, as follows from the IC condition (Definition 2).

Additionally, it is highlighted that the proposed model achieves almost the exact same social welfare for the overall examined system under the complete and incomplete information scenarios, where the incomplete information scenario concludes to a social welfare reduced only by 1% compared to the complete information scenario. To the best of our knowledge, this is the best achieved social welfare compared to the complete information scenario in the current bibliography. This novelty stems from the introduction of the Bayesian trust belief in the overall designed framework.

**Figure 5:**
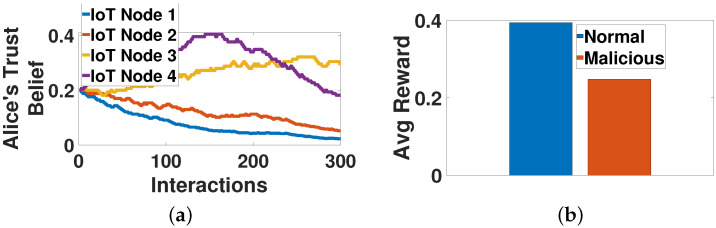
Behavioral change evaluation. (**a**) Alice’s belief vs. interactions, (**b**) average reward vs. behaviors.

In Figure 5, we present a scenario, where one IoT node (ID 4) starts behaving maliciously in the crowdsourcing process (e.g., investing small effort) at a specific time slot (t≈150), in order to examine the sensitivity of the proposed framework. Figure 5a shows that Alice senses the node’s change of behavior by experiencing a decreasing Bayesian trust belief regarding this node. Thus, Alice provides a lower average reward over time to this node compared to the scenario where the node presents normal behavior (Figure 5b), i.e., invests an effort as derived from the optimization problem **P2**.

### 5.3. Comparative Evaluation

**Figure 6:**
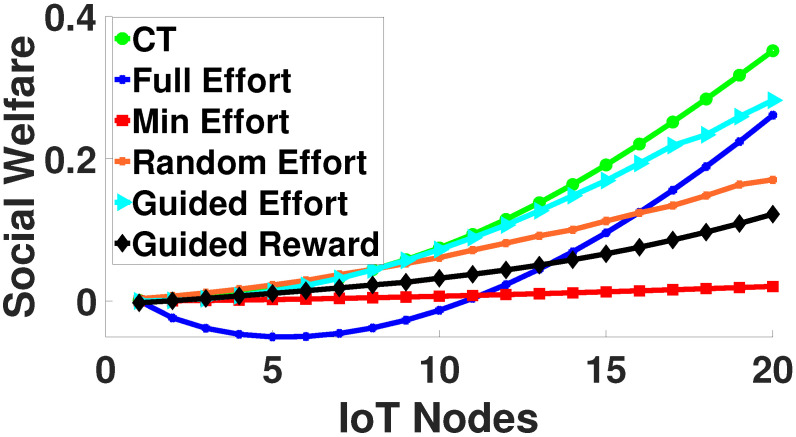
Offline contract-theoretic crowdsourcing—a comparative evaluation.

In this section, we present a comparative evaluation for $\left|C_{A}^{t}\right|=20$, considering six comparative scenarios: (1) the proposed offline contract-theoretic (CT) crowdsourcing, (2) Full Effort, (3) Min Effort and (4) Random Effort, where the nodes invest their maximum, minimum, and random effort, respectively, (5) Guided Effort, where the nodes invest $p_{c}^{t}\cdot \frac{\sum_{t\in [1,300]}r_{A,c}^{t}}{300},\forall c\in C_{A}^{t}$ effort, and (6) Guided Reward, where Alice provides a guided $\rho_{A,c}^{t}\cdot e_{A,c}^{t},\forall c\in C_{A}^{t}$ reward to each node. Figure 6 presents the cumulative social welfare as a function of the node’s ID.

The results reveal that the proposed framework enables the overall examined system to achieve the highest social welfare due to the joint exploitation of the Bayesian trust model and contract-theoretic crowdsourcing, which facilitate the intelligent inference of the nodes’ trust levels and their personalized treatment to invest their efforts in the crowdsourcing, respectively. The Guided Reward and Guided Effort scenarios present better social welfare compared to the myopic decision-making scenarios of Full, Min, and Random Effort, in terms of deciding the level of effort that the IoT nodes’ invest in the crowdsourcing process.

## 6. Conclusions

In this paper, an offline contract-theoretic crowdsourcing framework is introduced to enable two IoT nodes, Alice and Bob, to build a secure channel based on supporting information provided by selected nodes in the local ad-hoc network. Alice selects the IoT nodes by following a stochastic learning automata approach via exploiting the network characteristics and node trust levels. The trust is quantified by developing a PeerTrust model and exploiting the concept of Bayesian trust belief. A contract-theoretic approach is modeled among Alice and the selected IoT nodes, where Alice provides personalized rewards to the nodes in order for the latter ones to invest their effort in the crowdsourcing process and enable Alice to securely interact with Bob.

A detailed set of numerical and comparative results is provided to illustrate the operation, performance, and benefits of the proposed framework. The results suggest that the proposed offline contract-theoretic crowdsourcing framework achieves similar social welfare for the examined system under complete and incomplete information regarding the IoT nodes’ trust levels. Part of our current and future work focuses on the deployment of the model to a real-world scenario with practical IoT network data as well as on the extension of the presented model based on the theory of Satisfaction Games to capture the satisfaction-aware resource management in terms of collecting information from the selected nodes towards facilitating the system’s resource-saving and latency improvement, as envisioned with the Tactile Internet.

## Figures and Tables

**Table 1:** Summary of Key Notations.

Notation	Description
*t*	time slot
*C*	Set of IoT nodes
*c*	IoT node
*A*	Alice
$C_{A}^{t}$	Set of IoT nodes selected by Alice
$d_{c}^{t}$	Alice’s distance from an IoT node *c*
$cr_{c}^{t}$	Normalised congestion of the communication link between Alice and an IoT node *c*
$e_{A,c}^{t}$	Effort that Alice collects from the IoT node *c*
$e_{A,c}^{t*}$	Optimal effort
$r_{A,c}^{t}$	Personalized reward that Alice provides to an IoT node *c*
$r_{A,c}^{t*}$	Optimal reward
$\mu_{A,c}^{t}$	Bayesian trust belief of Alice regarding an IoT node *c*
$\mu_{0}$	Initial belief distribution
$a_{h}$	Probability that an IoT node provides high contribution
$a_{l}$	Probability that an IoT node provides low contribution
$S_{c}^{t}$	Number of times that an IoT node *c* contributed in a satisfactory manner up to time slot *t*
$F_{c}^{t}$	Number of times that an IoT node *c* contributed in a unsatisfactory manner up to time slot *t*
$p_{c}^{t}$	Score of an IoT node *c*
T(c)	Trustworthiness of an IoT node *c*
I(A,c)	Number of interactions that an IoT node *c* has with Alice
$TF_{A,c}$	Interaction context factor
α	Normalized weighting factor
$U_{c}^{t}\left(e_{A,c}^{t}\right)$	Payoff function of an IoT node *c*
$q(r_{A,c}^{t})$	Evaluation function of the received reward $r_{A,c}^{t}$
$U_{A}^{t}\left(e\right)$	Alice’s payoff function *o*
λ	Alice’s cost to provide rewards to the IoT nodes
$\rho_{c}^{t}$	Alice’s probabilistic estimation of an IoT node’s *c* score
SW(e)	Social Welfare
$A_{s}^{t}$	Alice’s discrete action space
$S^{t}$	Set of subsets of the |C| IoT nodes with cardinality $|C_{A}^{t}|$
ite	RL iteration
$F_{A,A_{s}^{t}}^{\left(ite,t\right)}$	Alice’s RL personalized feedback
${\hat{F}}_{A,A_{s}^{t}}^{\left(ite,t\right)}$	Alice’s RL normalized personalized feedback
${\mathrm{Pr}}_{A}^{\left(\mathrm{ite},t\right)}$	Alice’s action probability vector
*b*	RL learning parameter

**Table 2:** Simulation parameters.

Parameter	Value	Parameter	Value
|C|	10	$|C_{A}^{t}|,\forall t$	4
*b*	0.15	$\mu_{0}$	0.2
$a_{h}$	0.51	$a_{l}$	0.49
$S_{c}^{0}\forall c\in C$	1	α	0.8
$F_{c}^{0},\forall c\in C$	1	λ	0.7
$d_{c}^{t},\forall c\in C,\forall t$	[10 m, 400 m]	$TF_{A,c}^{t},\forall t$	0.5

## Data Availability

Not applicable.
